# Sandy Foundations

**Published:** 1883-11

**Authors:** N. W. Williams

**Affiliations:** Geneva


### Sandy Foundations.

BY DR. N. W. WILLIAMS, OF GENEVA.

Read at the American Dental Society of Cologne, Europe, Aug. 7th, 1383.

When one builds a house he must dig deep, and lay well and strong the foundation stones if he wants his house to stand the storms, and resist the destroying elements of time. If he builds his house upon the sands, the floods and winds may come and beat upon that house, and it will fall and great will be the fall. This fact holds good in dental operations on the teeth. The elements of destruction are ever present to destroy our superstructure, and what folly it would be to build upon the sand. The beautiful pearls that God has given us we find are soon, alas too soon, the subject of destruction, and dental art has come into existence to defeat the destroyer's aim. The art of filling teeth has been discovered for saving those precious gems. As members of such an important profession, with such momentous interests confided to our care it stands us in hand to devise and practice that system which brings about the best results, and saves the most teeth, and as a consequence produces the most happiness to mankind.

We are not only workers but teachers, and we should not only be careful how we work, but what we teach. Men in every profession and calling in life are prone to ride a hobby, and sometimes the tables are turned, and the hobby rides them. When we find one riding a hobby which is distined to do injury to the cause, it is our duty as members of a liberal profession to warn him and those he is trying to teach.

I believe I am echoing the sentiments of most of the profession when I say that gold has been found to be the best material yet discovered for saving teeth, other materials having their proper and useful places. But it has also been found to be the most difficult material to work properly, and requires a talent that unfortunately all who enter, or are in our profession do not possess. I do not believe any one has yet discovered an easy way of filling teeth properly with gold, although some operators do it more easily than others. I believe it our duty to try for our own comfort and that of our patients to make every effort to perform every operation as quickly and as painlessly as possible, having in view all the time the preservation of the tooth, and future health and comfort of the patient. We should hail with delight every advance step in that direction, and fight with vigor everything advanced, which is calculated to lead the younger members more especially astray, and which would lower the standard of our operations. Filling teeth with gold may be considered an invention of the present century. Our fore-fathers, in dentistry had to be content with filling teeth with non-cohesive gold foil, and necessarily were confined to filling and saving the most simple cavities. But, when cohesive gold was discovered the profession began to save teeth with its use, which had been up to that time consigned to the forceps. But with the advent of cohesive gold we are able to save thousands of teeth that otherwise would be lost or filled with unsightly amalgam. A dentist that prides himself in saving all teeth with gold is able to accomplish with cohesive gold, what is out of the question, with non-cohesive. But to say that some class of operations cannot be performed as well with non-cohesive gold would be nonsense, but for strength and wear in exposed places, and for contour the cohesive is necessary. To say that bad work has been made with it is just as true as to say that bad work is made with every material used in saving teeth. This results from bad manipulation or the character of the teeth worked upon. I believe many bad fillings have been made with cohesive gold by the use of too deeply serrated pluggers. With such instruments, the gold is cut up to such an extent as to leave the filling more or less porous and full of pits, so that after some time, if the filling is examined, it will show plain marks of the instruments. When this happens on the grinding surface it is not of so much importance, but when it is at the cervical wall and between the teeth, the result is often disastrous. In the use of cohesive gold, the object should be to drive the gold against the walls of the cavity, layer upon layer, and as smoothly against the walls, and each layer against its fellow, as it is possible to make them. Most of the instruments in use are calculated to defeat that object for the form and depth of the serrations is inclined to draw the gold from the walls. In my judgment the form of a point should be oval or beveled, or fuller in the center, so that when the point is pressed upon the gold, the strongest depression should be made in the center of the piece, and the condensation should be followed up from that point to the margin of the piece; in so doing the gold is better and more evenly condensed and driven against the walls, while if the points and serrations are sharp and deep and flat, the gold is condensed unevenly, and left full of pits. The gold should also be in ribbons or flat smooth surfaces, rather than in ropes or unevenly folded pieces. I have had some points made by the S. S. White Dental Manufacturing Co., from the form of the No. 16 of the C. R. Butler set, from that size down to as small as possible. After some months of use, I am much pleased with the result, as I find the gold works better to the borders, and they leave a better surface , which admits of a better finish, and is more quickly done. With the cavities carefully prepared, avoiding hills and hollow, as much as possible, and with the margins smooth, and with cohesive gold, and oval, and slightly serrated points to our pluggers, we are able to make as perfect a filling as it is possible to make with the present light we have.

